# Biochemical Studies on Methylglyoxal-Mediated Glycated Histones: Implications for Presence of Serum Antibodies against the Glycated Histones in Patients with Type 1 Diabetes Mellitus

**DOI:** 10.1155/2013/198065

**Published:** 2013-08-07

**Authors:** Nadeem A. Ansari, Debabrata Dash

**Affiliations:** ^1^Department of Biochemistry, Institute of Medical Sciences, Banaras Hindu University, Varanasi 221005, India; ^2^Department of Biochemistry, Universal College of Medical Sciences, Paklihawa Campus, Bhairawaha, Nepal

## Abstract

Reactive carbonyl species (RCS) mainly reacts with lysine and arginine residues of proteins to form advanced glycation end products (AGEs). Histone was glycoxidated with glyoxal and methylglyoxal. It was characterized by polyacrylamide gel electrophoresis and quenching studies involving penicillamine and aminoguanidine as carbonyl scavengers. Further characterization of histone modified with methylglyoxal was done by UV, fluorescence, and IR spectrophotometry. Spectral analysis of the protein clearly demonstrates structural perturbation in the histone by methylglyoxal. Methylglyoxal-induces cross-linking in the protein leading to aggregation. Role of methylglyoxal mediated glycoxidation of histone in type 1 diabetes was also undertaken. Antibodies were detected against glycoxidated histone in sera of type 1 diabetes patients by solid-phase enzyme immunoassay. The findings indicate that as a result of structural perturbation in histone by methylglyoxal, the modified histone may be involved in production of serum antibodies in the diabetes patients.

## 1. Introduction

There is overwhelming evidence for involvement of reactive oxygen species (ROS) in a number of pathophysiological conditions such as diabetes, cancer, and aging but the studies linking reactive carbonyl species (RCS) to the conditions are limited [1, 2]. RCS, such as methylglyoxal, is produced by degradation of lipid peroxidation products, early protein glycation adducts, and as a byproduct of glycolysis. RCS modification of histone results in cross-linking of proteins and induces ROS-dependent cleavage of plasmid DNA [3]. The proteasome degradation of RCS products is not complete and remnants may accumulate and cause epigenetic changes as well as further DNA and protein damage [4]. Earlier studies have shown that histones from liver cells of diabetic rats contain high level of AGEs [5]. We [6] have found antigenicity of glycated poly-L-lysine in experimental animals and autoantibodies were also detected against the modified lysine polypeptide in diabetes patients. A recent work has demonstrated in vivo formation of RCS-mediated AGEs in histone H_1_ using antibodies against oxidative protein adducts [7]. This study characterizes methylglyoxal-modified histone (a lysine-rich protein) and evaluates its role in type 1 diabetes patients.

## 2. Materials and Methods

### 2.1. Chemicals

Calf thymus whole histones (type II-A) and methylglyoxal were purchased from Sigma (St. Louis, MO, USA). Polystyrene flat bottom UV microtiter plates were obtained from Greiner BioOne (Germany). All other chemicals and reagents used in the study were of the highest analytical grade.

### 2.2. Serum Samples

Serum samples of various type 1 diabetes patients proven with diagnostic tests were obtained from Sir Sunderlal Hospital, IMS, BHU, Varanasi, India. These samples were routine determinations and were not specifically obtained for the study. Serum samples from normal healthy subjects who gave prior informed consent were used as control. The study has been approved by the ethics committee. 

All serum samples were decomplemented at 56°C for 30 min before use. 

### 2.3. Preparation of Methylglyoxal-Modified Histone

Histone was glycoxidated as described earlier with minor modifications [3, 8]. Briefly, histone (1 mg/mL) in phosphate-buffered saline (PBS, pH 7.4) was modified by separate incubation with glyoxal (1 and 2 mM) and methylglyoxal (1 and 2 mM) in 0.25 M sodium phosphate buffer, pH 7.4, at 37°C for 24 h. Unmodified histone dissolved in the same buffer served as control. Low concentration of RCS is taken as it binds preferentially to lysine and arginine-rich histones and rapidly forms dimers and polymers [9].

### 2.4. Effect of Carbonyl Scavengers on Methylglyoxal-Modified Histone

Effect of scavengers on RCS modification of histones was determined by addition of coincubated mixtures of penicillamine/aminoguanidine with methylglyoxal or glyoxal to histones and incubation of the reaction mixture at 37°C for 24 hr. At the end of incubation, fluorescence of the assay mixture was read after excitation at 320 nm and percent quenching was calculated. 

### 2.5. Polyacrylamide Gel Electrophoresis

Methylglyoxal-induced alteration in histone was examined by sodium dodecyl sulfate polyacrylamide gel electrophoresis (SDS-PAGE) as described previously on 15% gel under reducing conditions, followed by silver staining as described previously [3]. 

### 2.6. UV Spectrophotometry

The UV absorption characteristics of control and modified histone were recorded on UV-visible spectrophotometer (Biotek, model FLx800) between 200 and 400 nm using UV microtiter plates. The increase in absorbance (hyperchromicity) was calculated using the following formula:

(1) Hyperchromicity  (%)=(Amodified−AcontrolAmodified)×100.

### 2.7. Fluorescence Study

Control and modified histones were subjected to fluorescence studies on spectrofluorophotometer (Hitachi, model F-2500). Fluorescence was measured at 25 ± 0.1°C in cuvette of 1 cm pathlength, with slit width fixed at 10 nm. Formation of AGE-chromophores on histone was measured by AGE-type fluorescence at 320/380, 335/385 nm (*λ*_ex_/*λ*_em_). Increase in fluorescence intensity (FI) was calculated using the following formula:

(2)   %  Increase  of  FI=(FImodified−FIcontrolFImodified)×100.

### 2.8. FTIR Spectroscopy

The FTIR absorption spectra of the samples were recorded on Nicolet 5700 FTIR spectrometer (Thermo Corporation) in the range from 1500 to 1700 cm^−1^ with a nominal resolution of 4 cm^−1^. All measurements were carried out at room temperature. Automated spectra of control and modified histones were obtained from the subtraction spectra [(control + buffer) − (buffer)] and [(control + modified + buffer) − (control + buffer)].

### 2.9. Detection of Antibodies against Methylglyoxal-Modified Histone in Type 1 Diabetes Patients

Presence of antibodies against the modified histone in the sera of type 1 diabetes patients was evaluated by solid-phase enzyme immunoassay. Enzyme-linked immunosorbent assay (ELISA) was performed on polystyrene plates as described previously [10]. Absorbance (*A*) was monitored at 410 nm on an automatic microplate reader and mean of duplicate readings for each sample was recorded. Results have been expressed as a mean of *A*_test_ − *A*_control_. 

## 3. Results & Discussion

**Figure 1:**
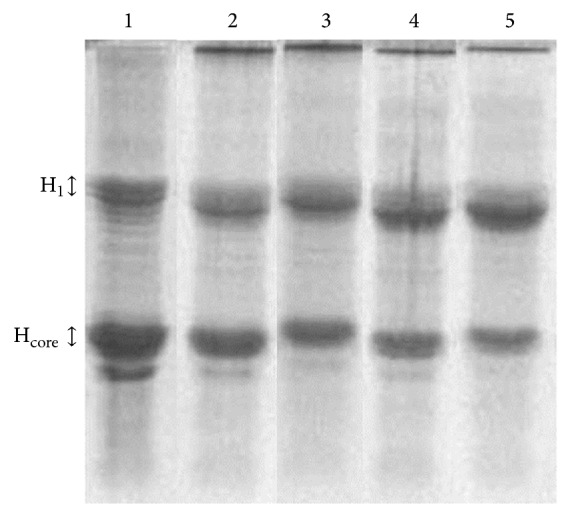
SDS-PAGE (15%) of native histone (lane 1) and histone modified with 1 and 2 mM methylglyoxal (lanes 2 and 3, resp.) and 1 and 2 mM glyoxal (lanes 4 and 5, resp.). Electrophoresis was carried out for 3 h at 80 volts and the gel was visualized using silver staining.

RCS-induced alteration in histone was examined by sodium dodecyl sulfate polyacrylamide gel electrophoresis (SDS-PAGE) on 15% polyacrylamide gel after silver staining. The migration pattern of silver-stained bands during SDS-PAGE is shown in Figure 1. The pattern of native histone revealed sharp bands of linker and core fractions (lane 1) which underwent significant changes upon modification with 1 and 2 mM methylglyoxal (lanes 2 and 3, resp.) and 1 and 2 mM glyoxal (lanes 4 and 5, resp.). Both of the modified histones exhibited slightly higher-molecular-weight core histone fractions (lanes 3 and 5), having reduced mobility in the gel compared to the native ones. The modifier produced aggregation of histone molecules into high molecular weight polymers, bands of which were restricted to top of the stacking gel. A more extensive histone polymerization caused by methylglyoxal, with a 2 mM concentration, showed that it was a slightly more effective protein cross-linker. Glycation-mediated cross-linking of proteins is known to produce dimers and high-molecular-weight polymers having reduced mobility in electrophoresis [11]. 

**Figure 2:**
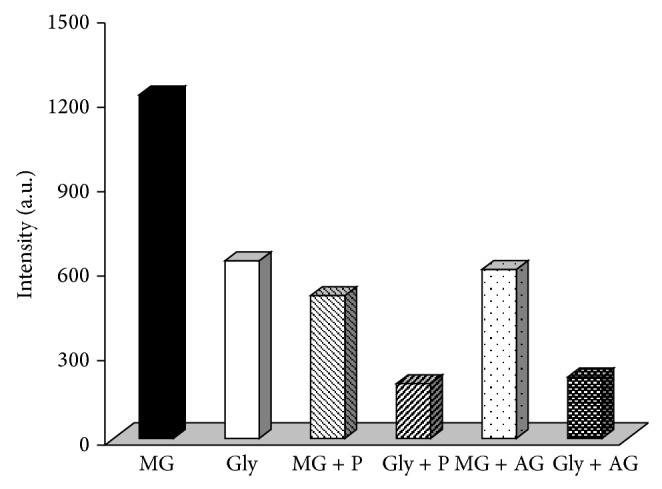
Fluorescence inhibition by penicillamine (P) and aminoguanidine (AG) during incubation of histones with methylglyoxal (MG) and glyoxal (Gly).

 Penicillamine and aminoguanidine used in the present study are potent carbonyl scavengers and exert a direct effect on RCS. When the scavengers (3 mM) were present in the RCS-modified reaction mixture, the corresponding results were shown in Figure 2. They showed significant inhibitory effect on the fluorescence induced by both methylglyoxal- and glyoxal-modified histones. There was quenching of 58% and 69% in fluorescence intensity of methylglyoxal- and glyoxal-modified histones by penicillamine while it was 51% and 65% by aminoguanidine. This study confirmed involvement of the RCS in glycoxidation of histones and indicate that methylglyoxal was a more effective modifier than glyoxal.

**Figure 3:**
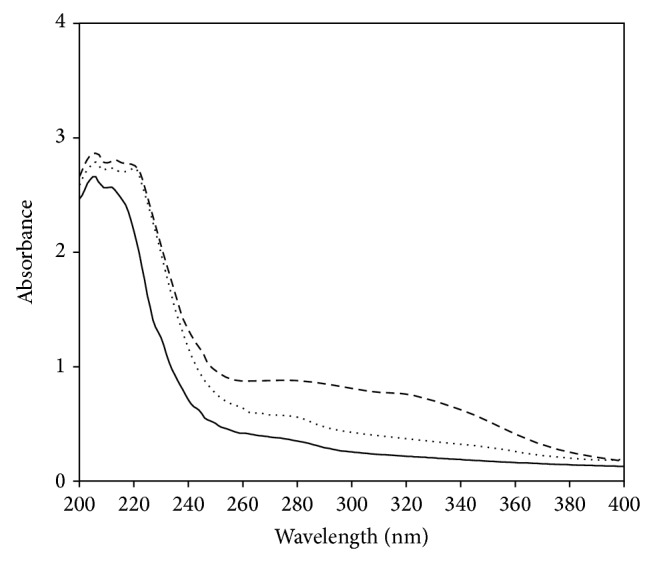
Absorption spectra of native histone (—) and histone modified with 1 mM (⋯) and 2 mM (- -) methylglyoxal.

Histone modified with different concentrations of methylglyoxal was further characterized by spectral techniques. UV and fluorescence data of histone modified with methylglyoxal are summarized in Table 1. Native histone had characteristic absorption peak at 206 nm due to its lysine content (Figure 3). Upon modification with methylglyoxal, increase in absorbance was recorded, extent of which was dependent on the modifier concentration. Modified histone samples also exhibited new broad peak at 320 nm. AGE adducts of lysine and arginine residues in glycated proteins are determined by AGE-specific absorbance at 330 nm [12]. The hyperchromicity exhibited by histone, modified with 1 and 2 mM methylglyoxal, were 41% and 76%, respectively, at 320 nm. The increase in absorbance and appearance of AGE-specific peak in histone modified with the RCS is an indication of structural changes in the histones. 

**Figure 4:**
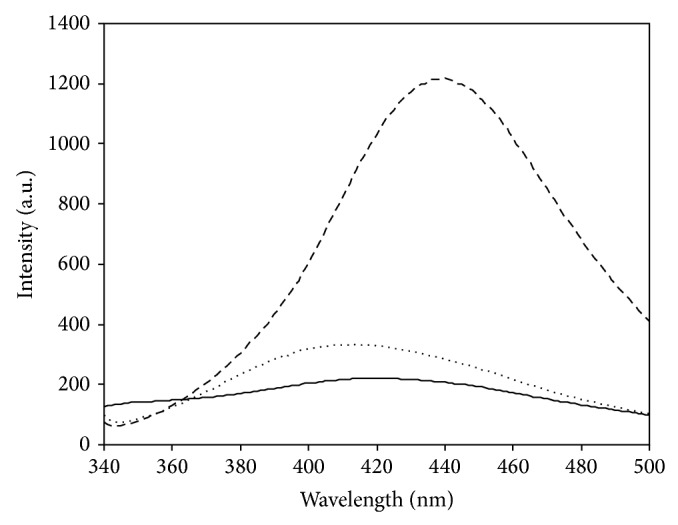
Fluorescence emission spectra of native histone (—) and histone modified with 1 mM (⋯) and 2 mM (- -) methylglyoxal. Excitation, 320 nm.

AGE formation in methylglyoxal-modified histone led to an increase in fluorescence intensity, associated with shift in the emission maximum from 419 nm (native histone) to 413 and 440 nm in presence of 1 and 2 mM methylglyoxal, respectively, upon excitation at 320 nm (Figure 4). The observed increase in intensity was 34% at 413 nm (1 mM methylglyoxal) and 82% at 440 nm (2 mM methylglyoxal). However, the intensity enhancement was 26% at 419 nm and 74% at 438 nm with 1 and 2 mM methylglyoxal (data not shown), upon excitation at 335 nm. This observation may be attributed to rise in the level of AGE adduct in histone induced by increasing concentration of RCS. The emission wavelength between 405–410 nm has been shown to reflect AGE structures of argpyrimidine and pentosidine [12]. 

**Figure 5:**
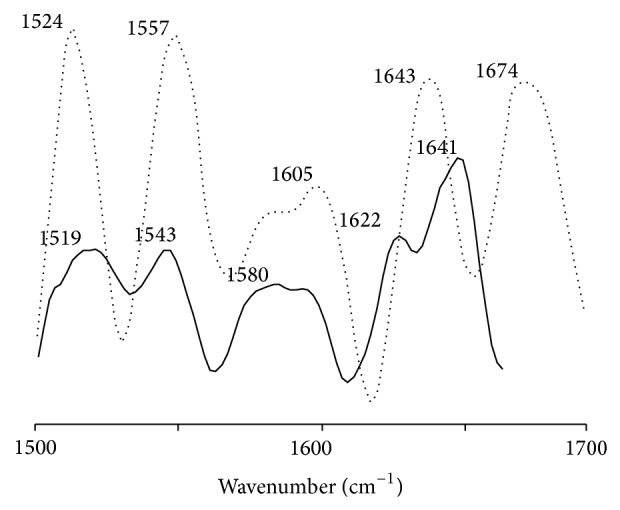
FTIR spectra of histone (—) modified with methylglyoxal (⋯).

The conformational changes in RCS-modified histone were determined by FTIR spectra (Figure 5). There was shift in position and intensity of amide bands with respect to native histone at pH 7.4. Amide I gives rise to infrared bands in the region between 1600 and 1700 cm^−1^ and is considered a sensitive probe to study secondary structures of proteins, namely, random coils, *α* helix, and *β* sheets. The main spectral features of native histone were characterized by an intense band at 1641 cm^−1^ and a weak one at 1622 cm^−1^ corresponding to *α* helix and pleated sheet structures, respectively. In contrast, histone modified with a 2 mM of methylglyoxal showed distinct variations in both peak position and intensity, suggestive of changes in the histone structure upon modification. The bands at 1641 and 1622 cm^−1^ were shifted to 1643 and 1605 cm^−1^ in methylglyoxal-modified histone. The prominent and broad peak bands in the range of 1605–1610 cm^−1^ indicate a higher content of pleated structure in the modified histones whereas the appearance of a strong band in the range of 1670–1675 cm^−1^ in the modified histones corresponded to the presence of random form. A similar change in both peak position and intensity of histone modified with RCS appears in amide II bands in the region between 1500 and 1600 cm^−1^. The diagnostic amide I IR bands corresponding to native and modified histones are compiled in Table 2.

**Figure 6:**
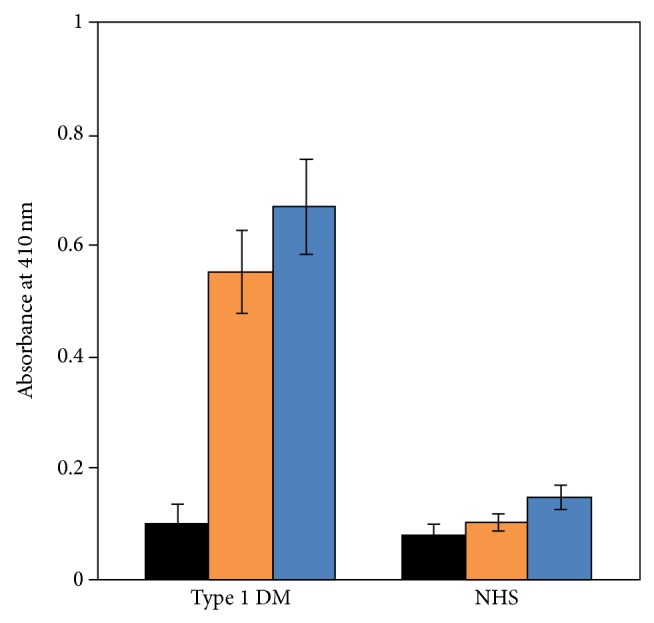
Direct binding ELISA of 1 : 100 diluted type 1 diabetes mellitus serum (type 1 DM) and normal human serum (NHS) samples to native histone (black square) and histone modified with 1 mM (orange square) and 2 mM (blue square) methylglyoxal.

We have determined role of the modified histone in type 1 diabetes. This study comprised 22 serum samples from the diseased patients while 11 samples were from healthy controls. Serum antibodies from diabetes patients showed appreciable binding to methylglyoxal-modified histone as compared to the native form at 1 : 100 serum dilution (*P* < 0.001) in direct binding ELISA (Figure 6). Binding increased with rise in concentration of the modifier as revealed by absorbance studies based on ELISA. The average absorbances (±SD) with the sera of type 1 diabetes patients binding to native histone and histone modified with 1 and 2 mM methylglyoxal were 0.15 ± 0.03, 0.56 ± 0.07, and 0.67 ± 0.08, respectively. No appreciable binding was observed in normal subjects. We have found antibodies to AGE modified IgG, a lysine-rich protein, in type 1 diabetes patients [13]. Another study has shown promising result towards detection of both lysine-derived and arginine-derived AGEs by the induced antibodies against AGE-modified BSA [14]. There is involvement of nonenzymatic glycosylation of proteins, specifically on lysine residues, in age-related diseases [15, 16]. Recent studies have implicated lysine in inhibition of the glycation process both in vitro and in vivo [17, 18]. 

## 4. Conclusions 

The results reported here demonstrate that spectral analysis of methylglyoxal-modified histone provides a useful insight into the structural perturbation of lysine and arginine residues in histone and determines formation of AGE adducts in histone during the 24 h incubation of histone with methylglyoxal. Methylglyoxal-mediated histone glycation is also recognized by serum antibodies of type 1 diabetes patients. It has been revealed from our study that the RCS-modified histones might be a potential target for circulating autoantibodies in patients with type 1 diabetes mellitus and this target can be used as a predictive biomarker for controlling the disease complications.

## Figures and Tables

**Table 1:** UV and fluorescence data of native and methylglyoxal modified histones under identical experimental conditions.

	Native histone	Methylglyoxal-modified histone	Modification
UV absorbance at 320 nm^a^	0.22	0.37	41.2%
UV absorbance at 320 nm^b^	0.22	0.76	76.2%
Fluorescence intensity^a^ (Ex. 320 nm)	222 (Em. 419 nm)	335 (Em. 413 nm)	33.7%
Fluorescence intensity^b^ (Ex. 320 nm)	222 (Em. 419 nm)	1220 (Em. 440 nm)	81.7%

a and b represent 1 and 2 mM concentrations, respectively, of the histone modifier.

**Table 2:** Prominent FTIR amide I bands in secondary structure of histone modified with methylglyoxal (MG).

	Random coil	*α* Helix (in cm^−1^)	*β* Sheet
Histone^n^	—	1641	1622
MG-modified histone^a^	1674	1643	1605

n: native histone.

a represents 2 mM concentration of the histone modifiers.
